# Cultivation of Intrapreneurship: A Framework and Challenges

**DOI:** 10.3389/fpsyg.2021.731990

**Published:** 2021-10-18

**Authors:** Lan-Ying Huang, Shu-Min Yang Lin, Ying-Jiun Hsieh

**Affiliations:** ^1^Department of Business Administration, National Changhua University of Education, Changhua City, Taiwan; ^2^Graduate Institute of Technology Management, National Chung Hsing University, Taichung, Taiwan

**Keywords:** intrapreneurship, corporate entrepreneurship, innovation, sustainability, integrated framework

## Abstract

Intrapreneurship has drawn research attention over the past decades considering its crucial role in innovation, organizational performance, and employee career planning. Intrapreneurial research based on various concepts also emerges. In spite of the increasing concern in the field, contributions in the field are fragmented. Particularly, intrapreneurship research is still lacking an integrated framework based on which, enablers and important facilitating mechanisms can be identified to enhance intrapreneurship. To close the above research gap, the study develops a holistic intrapreneurial framework. Specifically, the study first examines intrapreneurship in relation to other prominent concepts (i.e., innovation, entrepreneurship, and sustainability). This study then identifies enablers of intrapreneurship at both individual and organizational level. Notably, extant research largely examines intrapreneurship either at the organizational or individual level, and concentrates in corporate entrepreneurship or individual intrapreneurial employees. Research providing a holistic perspective on enablers for intrapreneurship is rare. The study further integrates these intrapreneurial enablers with facilitating mechanisms and proposes a framework of intrapreneurship. The framework makes it possible to clearly identify pivotal antecedents to intrapreneurship based on various theoretical lenses and analytical levels applied. Finally, the study addresses a list of managerial and technological challenges arising from the above framework and suggests future research agenda.

## Introduction

Entrepreneurship may occur within an organization or firm, that is, intrapreneurship (Corbett et al., 2013). Prior research suggests that intrapreneurship helps firms innovate, enhance internal performance, adapt to external changes, and reinvigorate their businesses (Augusto Felício et al., 2012). Research also indicates that nurturing employees’ intrapreneurial engagement leads to permanent organizational development (Falola et al., 2018). Namely, involvement, empowering employees, job autonomy, and appropriate reward system allow employees to produce innovative ideas and pursue innovation opportunities which strengthen firms’ long-term strategic performance (Adeyeye et al., 2015). Academic interests in intrapreneurship increase over the last decade with a diverse focus (Chan et al., 2017; Alam et al., 2020b). Intrapreneurship in practice, however, is not as wide-spread as expected. The phenomenon of intrapreneurship also differs geographically. Recent research indicates that the Nordic countries lead with a prevalence of intrapreneurship around 9%, whereas the opposite is observed for Mediterranean and Eastern European countries as well as developing countries (Elert et al., 2019). The observation that growing academic interests in intrapreneurship does not reflect proportionately in practice may attribute to practitioners’ concerns about, in particular, how to structure an appropriate internal process to facilitate intrapreneurship within the firm.

While intrapreneurial research continues to bridge the gap between theory and practice, several research gaps still remain in the literature. For example, over the past decades, the term intrapreneurship appears occasionally in the context of innovation, entrepreneurship, or sustainability as emerging business concerns for approaching excellence. How are these pivotal theoretical perspectives relating to each other? Answering this question shall lead to desired business performance. Furthermore, intrapreneurial studies largely hold a static view and describe its phenomenon, measurements, impacts, various forms, etc. (e.g., Gawke et al., 2019). The study urges that intrapreneurial research needs to become more a kaleidoscope than a mosaic, and starts to recognize the dynamic nature of intrapreneurship, namely, considering critical, correlated factors, and facilitators at the same time. In other words, how to cultivate intrapreneurship within a firm from a holistic perspective? The study aims to answer the above two questions as insufficient research efforts were found to address these important concerns.

**FIGURE 1:**
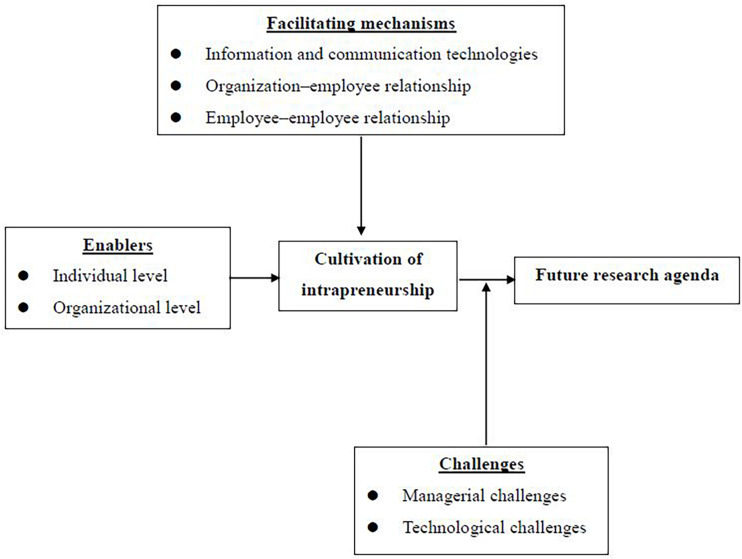
Conceptual framework for cultivation of intrapreneurship.

The study is organized as follows. The next section examines intrapreneurship in relation to other prominent concepts (i.e., innovation, entrepreneurship, and sustainability) in business development and management. The study then elaborates on the various enablers at both individual and organizational levels, as well as facilitating mechanisms that foster intrapreneurship for businesses. The study further demonstrates the potential challenges arising from these antecedent conditions and suggests an agenda for future studies. The final section provides conclusions as well as theoretical and managerial implications. Figure 1 demonstrates the conceptual framework with the key elements.

## Intrapreneurship Through Various Theoretical Lens

Antoncic (2007) defines intrapreneurship simply as entrepreneurship within an organization. Corporate entrepreneurship (CE) also means intrapreneurship (Sharma and Chrisman, 1999; Rigtering and Weitzel, 2013). Or, intrapreneurship is a manifestation of CE (Sharma and Chrisman, 1999). Though scholars often adopt the terms CE and intrapreneurship interchangeably, several studies suggest that CE represents entrepreneurial activities which are initiated top-down within the firm, whereas intrapreneurship implies entrepreneurial activities pursued bottom-up by the firm’s employees (e.g., Rigtering and Weitzel, 2013). Research suggests that the dimensions of intrapreneurship include: (1) new business venture, i.e., entering new dimension with the organization’s products and service, (2) inventiveness, i.e., finding new products, services and technologies and commercialization process, (3) organizational renewal, i.e., alteration, development and restructuring, and (4) proactivity, i.e., acting in advance to deal with an expected difficulty (Antoncic and Hisrich, 2001). Intrapreneurship manifests itself in a multi-dimensional nature that is based on diverse theoretical frameworks of entrepreneurship. These frameworks take root in innovation entrepreneurship (Schumpeter, 1934) or innovation management (Drucker, 1979). Particularly, intrapreneurial research stems from the need to develop a sustainable approach to achieve innovations within a firm (Covin and Slevin, 2002). The terms intrapreneurship, innovation, entrepreneurship, and sustainability, though seemingly different, do share a number of commonalities. Their convoluted relationships are discussed as follows.

### Intrapreneurship and Innovation

Augusto Felício et al. (2012) suggest that intrapreneurship can be explained by innovation and several other pivotal factors. Researchers link intrapreneurship with innovation mainly from the following perspectives: definition, process, and outcomes. From the perspective of definition, Vesper (1984) defines intrapreneurship as “employee initiative from below in the organization to undertake something new; an innovation which is created by subordinates without being asked, expected, or perhaps even given permission by higher management to do so” (p. 295). Carrier (1996) indicates that intrapreneurship is the same as innovation that is initiated bottom-up by employees. Covin and Miles (1999, p. 49) state that “innovation is at the center of the nomological network that encompasses the construct of corporate entrepreneurship.” Later, Antoncic and Antoncic (2011) stress that intrapreneurship comprises the following key components: business self-renewal, new business ventures, and innovativeness related to product, service, process, or technology. Hence, intrapreneurship and innovation appear to represent two sides of the same coin.

From the perspective of process (i.e., employees and their behaviors), intrapreneurial employees often generate and pursue innovative ideas and information. Through sharing ideas and information with peers, the organization becomes more competitive and innovative. Specifically, research considers intrapreneurial employees and projects as important driving forces for a firm’s innovation and strategic renewal (Rigtering and Weitzel, 2013). Regarding intrapreneurial behavior, this term manifests itself in various dimensions such as employees’ creativity, proactivity, innovativeness, ability to explore/exploit business opportunity, as well as their networking and risk-taking behaviors (Neessen et al., 2019). Thus, intrapreneurial behavior can be perceived as a type of innovation-related outcomes. Research suggests that employees’ intrapreneurship relates positively to their voluntary searching and sharing of signaling information (i.e., scouting) (Park et al., 2014). Employees’ scouting behaviors are considered as strategic and may create value for firms. Through recognizing and disseminating crucial information for managerial and operational innovation, these behaviors also help the organization enhance its adaptiveness (Kim and Rhee, 2011). Furthermore, Skarmeas et al. (2016) assert that intrapreneurship can enhance a firm’s present and future performance via exploration and exploitation capabilities (i.e., innovations-driven dynamic capabilities), which develop new knowledge or improve existing knowledge about a market.

From the perspective of outcomes (e.g., innovation as intrapreneurial outcome), there exists several studies explaining why and how intrapreneurship drives innovation (e.g., how the intrapreneurial activities relate to a firm’s innovative performance) (Camelo-Ordaz et al., 2012). Intrapreneurship becomes increasingly popular among practitioners to foster innovation and exploit business opportunities within a firm, as well as enhance the firm’s innovative performance (Rigtering and Weitzel, 2013). Particularly, intrapreneurial efforts lead to various innovative results, for example, organizational renewal, creation of new businesses, and various product/process innovation (Vargas-Halabí et al., 2017). Likewise, intrapreneurial outcomes can be measured from the following perspectives: organizational self-renewal, new business ventures, and innovations (e.g., developing new products). Other exemplary outcome variables in intrapreneurship include long-term organizational performance and individual success (e.g., Rigtering and Weitzel, 2013; Baggen et al., 2016). In short, intrapreneurs can be viewed as employees who demonstrate innovative characteristics and behaviors at work, and thus achieve innovative outcomes for firms. Intrapreneurship, as a result, can be perceived as an innovation driven by employees’ spontaneous behaviors.

### Intrapreneurship and Entrepreneurship

Several studies probe into the similarities and differences between intrapreneurship and entrepreneurship, and how they relate to each other (e.g., Douglas and Fitzsimmons, 2013; Chahine, 2021). Intrapreneurship and entrepreneurship share several common characteristics such as innovation, creation of value, and undertaking risk (Cadar and Badulescu, 2015). Entrepreneurs often take advantage of their human and social capital to establish ventures, and sell products/services to external customers. In contrast, intrapreneurs usually initiate new business opportunities within their organizations (Parker, 2011). Furthermore, intrapreneurial activities have a higher success rate compared to regular entrepreneurial startups (Shah et al., 2014). Specifically, the intrapreneurial success rate can be as high as 80%, whereas the success rate for typical startups is around 20%.

With respect to individual characteristics, first, research finds that self-efficacy relates to both intrapreneurial and entrepreneurial intentions (Douglas and Fitzsimmons, 2013). Nonetheless, considering the principal outcomes for an individual, intrapreneurship and entrepreneurship indeed represent distinct entrepreneurial behaviors. Specifically, employees’ attitudes toward personal income, ownership, and autonomy at work are associated with their entrepreneurial intentions, whereas their attitudes toward risk are associated with intrapreneurial intentions. Second, intrapreneurs and entrepreneurs are both innovative persons. They aim to add further value to the products and services. Both the intrapreneurial and entrepreneurial activities engender a higher degree of risk compared to other regular activities in the firm. However, the intrapreneur risks the firm’s resources (e.g., funds etc.) aiming to develop new products and services, whereas the entrepreneur takes risks with his/her own financial, social, and human capital.

With respect to organizational deployment, research suggests that the entrepreneurial leadership model can be applied to the intrapreneurial context in the education sector, except for the risk-taking competency (Boon et al., 2013). Furthermore, entrepreneurial research proposes two main approaches to set corporate entrepreneurship within a firm: focused and dispersed (Birkinshaw, 1997). The focused approach [e.g., new venture division (NVD), internal corporate venturing, etc.] assumes that intrapreneurial activities represent distinct work processes that should be separated from other regular managerial works within the firm. In contrast, the dispersed approach suggests a dual role for every employee, namely, both managerial and intrapreneurial behavior. The dispersed approach allows firms for sensing greater diversity of intrapreneurial opportunities as the intrapreneurial activities are deployed throughout the organization, rather than limited within an NVD. Under such setting, every employee is a potential bottom-up intrapreneur. Later, research examines the contributions of various managerial levels (i.e., top-, middle-, and operating-level) to the CE process at different stages (i.e., discovery, evaluation, legitimation, and exploitation) and their exchanges between each other (Belousova and Gailly, 2013). Indeed, quite a few studies emphasize the pivotal role of middle-level managers’ role in encouraging intrapreneurship (Kuratko et al., 2005; Blanka, 2019). As such, middle-level managers advocate, modify, and guide intrapreneurial opportunities and further determine, obtain and allocate necessary resources to pursue these intrapreneurial opportunities (Blanka, 2019). In other words, middle-level managers support corporate entrepreneurial initiatives broken down from top level executives and promote their value creating potential to operational-level managers, namely, the primary executors (Kuratko et al., 2005). Still, organizations pursuing CE strategies are likely to demonstrate a stratified but integrated series of intrapreneurial actions at the top, middle, and operational-levels of management; managers across organizational levels take joint responsibility for the success of these intrapreneurial actions (Hornsby et al., 2009). Hence, the focused approach resembles the intrapreneurial deployment in a top-down manner whereas the dispersed approach typifies the bottom-up intrapreneurial process.

Together, intrapreneurs can be perceived as internal entrepreneurs who take risks (though different to the type of risks typical entrepreneurs may face), add values to their work, and contribute to product/service innovations, new business ventures, or strategic self-renewal within the firm. Intrapreneurship is thus entrepreneurship within an existing organization with a bottom-up, instead of top-down focus. Furthermore, managers across levels play specific roles in the intrapreneurial process.

### Intrapreneurship and Sustainability

Business sustainability focuses on what needs to be sustained (e.g., organizations) and what needs to be developed (e.g., individuals) (Shepherd and Patzelt, 2011). For businesses, sustainability is the ability to exist and develop without depleting further resources, namely, making use of existing resources, for the future. In this regard, it is not surprised that intrapreneurial research stems from the need to develop a sustainable approach to achieve innovations within a firm (Covin and Slevin, 2002). Focusing on innovation alone, however, is not sufficient for sustainability in the current hostile business environment. Intrapreneurship may relate to sustainability in multiple ways, namely, strategically or behaviorally.

From the strategic perspective, to achieve sustainable development, firms need necessary and specific resources and capabilities. For example, research considers intrapreneurship as a vital strategic resource that guides a firm’s principle of competition, and leads to sustainable firm performance (Urbano et al., 2013). From either the resource-based view (i.e., intrapreneurship as a strategic resource) or dynamic capabilities view (i.e., intrapreneurship as a firm-specific capability), studies aver that intrapreneurship acts a pivotal role for firms to attain sustainability (e.g., Skarmeas et al., 2016). In particular, Criado-Gomis et al. (2018) consider both intrapreneurship and sustainability as the internal organizational generative capability (i.e., the capability to reconfigure operational capabilities to address external changes and lead to organizational renewal), as Teece (2007) defines the term “dynamic capability.” Their study further shows that intrapreneurship indeed facilitates the process, through which, sustainable entrepreneurial orientation exerts positive effect on business performance.

From the behavioral perspective, research avers that employees’ intrapreneurial behaviors relate positively to their job performance (Ahmad et al., 2012). As such, creating a work environment that is conducive for employees to behave intrapreneurially helps foster innovative culture, which in turn leads to a firm’s sustainable long-term growth. Scholars examine how intrapreneurship in small and medium-sized enterprises (SMEs) attains sustainable innovation via process innovation. The results indicate that the elements of intrapreneurship such as proactiveness, risk taking and autonomy pave the way to sustainable economic, environmental and social innovation (Widya-Hastuti et al., 2016). Furthermore, Parris and McInnis-Bowers (2017) suggest that entrepreneurial activities within organizations, namely intrapreneurship, serves as a mechanism to bring forth profitable results for firms; it also provides valuable solutions for the society to achieve a sustainable future. The above discussion indicates that, through the lens of sustainability, intrapreneurs can be viewed as a critical human resource that firms need to develop to achieve sustainability. Intrapreneurship, from an organizational view, can be perceived as a sustainable strategic resource and dynamic capability, that firms can adopt to exploit or explore business opportunities.

**FIGURE 2:**
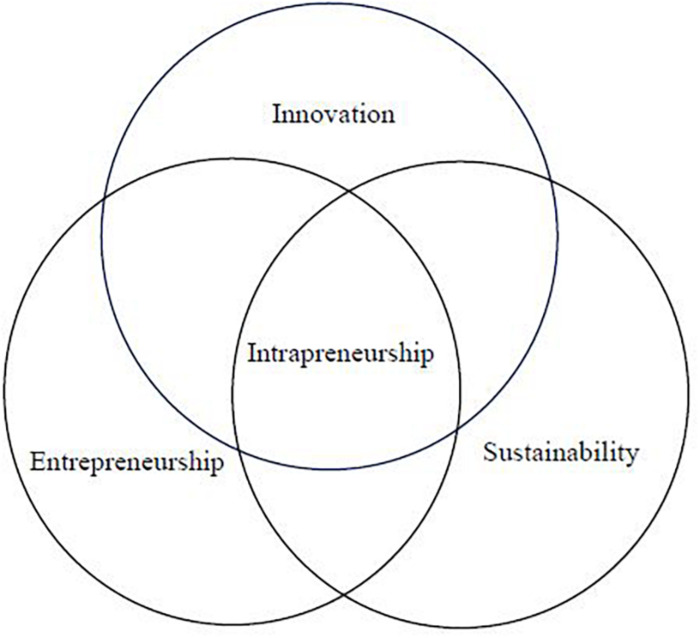
Venn diagram showing the concept of intrapreneurship shared by innovation, entrepreneurship, and sustainability.

To demonstrate the intertwined relationships among intrapreneurship, innovation, entrepreneurship, and sustainability, the study proposes the following Venn diagram of intrapreneurship: at the confluence of these three constituent theoretical perspectives (Figure 2). The study also summarizes the relationships between intrapreneurship and innovation, entrepreneurship, and sustainability, respectively in Table 1.

**TABLE 1:** Relationships between intrapreneurship and innovation, entrepreneurship, and sustainability.

**Intrapreneurship vs. innovation**	**Intrapreneurship vs. entrepreneurship**	**Intrapreneurship vs. sustainability**
***Definition***	***Individual characteristics***	***Strategic perspective***
• Intrapreneurship as employee initiative from below in the organization to undertake innovations • Innovation is at the center of the nomological network that encompasses the construct of intrapreneurship ***Process (employees and their behaviors)*** • Intrapreneurial employees and intrapreneurial projects are important drivers of innovation within companies • Intrapreneurial behavior manifests itself in innovativeness, creativeness, etc. ***Outcomes*** • Intrapreneurship as a tool to foster innovation and opportunity exploitation within a firm • Intrapreneurial efforts lead to innovative results: organizational renewal, creation of new businesses, and product/process innovation	• Self-efficacy • Risk propensity • Creation of value • Innovative ***Organizational deployment*** • Focused approach which works on the premise that entrepreneurship and management are fundamentally different processes and that they need to be separated structurally (e.g., new venture division) • Dispersed approach through which entrepreneurial initiatives are developed as embedded in the corporate context by the employees who combine the entrepreneurial activity with their usual job	• Intrapreneurship as a vital strategic resource that guides a firm’s philosophy of competition, and leads to sustainable firm performance • Dynamic capability view: Both intrapreneurship and sustainability are the internal organizational generative capability (e.g., the capability for operational renewal) ***Behavioral perspective*** • Forming an internal ecosystem that is conducive for the workforce to behave intrapreneurially helps foster innovative culture, which in turn leads to long-term growth and sustainability of the firm • The elements of proactiveness, risk taking and autonomy in intrapreneurship provides a leverage for sustainable business innovation • Intrapreneurship as a mechanism to generate profitable results for firms and valuable solutions for the firm, which lead to sustainable future

## Cultivating Intrapreneurship: Enablers and Facilitating Mechanisms

New ventures can commence within an organization if the organization encourages its employees to think intrapreneurially about their daily work. Fostering a supportive atmosphere for intrapreneurial engagement is crucial for employees to take initiatives toward business excellence and exploring business opportunities (Parker, 2011; Arnab, 2014). Several studies probe into the topic of cultivating intrapreneurship. For example, from the view of innovation, Sung and Choi (2011) propose four key agents of innovation, namely, top management, external environment, innovation and employees. Each agent plays particular roles in the various stages when implementing CE. Other studies, however, largely hold an entrepreneurial perspective or in combination with organization theories (e.g., Boon et al., 2013). Drawing on additional related theoretical view of sustainability, the study establishes a holistic framework to nourish intrapreneurship for businesses. The study categorizes and discusses three key elements of the framework, namely, individual enablers, organizational enablers, and facilitating mechanisms as follows.

### Enablers-Individual Level

As noted above, individual employees are capable of developing creative ideas to innovate organizational process from their daily work. Individuals who act as entrepreneurs within an existing organization are considered as intrapreneurs or corporate entrepreneurs. Intrapreneurs are often capable of identifying, screening, and exploiting business opportunities for the company to create value (Ma et al., 2016). Prior studies identify a list individual related factors that affecting intrapreneurship. The study summarizes these findings and proposes four major types of enablers at the individual level: self-attitudes, capabilities, judgments, and personality attributes/traits.

#### Self-Attitudes

From the bottom-up perspective, intrapreneurs act as individuals who possess the entrepreneurial spirit, initiating an upward process of change, or as teams that are proactive and take the lead to fulfill organizational goals for improvement and sustainable growth (Sinha and Srivastava, 2013). Specifically, research emphasizes the key role of personal initiative in engendering intrapreneurial behaviors (Gawke et al., 2019) and translating employees’ behavior into intrapreneurial projects (Rigtering and Weitzel, 2013). Likewise, Wakkee et al. (2010) aver that employee perceived entrepreneurial self-efficacy (i.e., person’s belief that he or she has the motive and resources, and is capable of successfully completing a task) leads to entrepreneurial behavior within the firm. Namely, an employee’s perception of his/her own capability to behave entrepreneurially can be demonstrated by the actual entrepreneurial behaviors (see also Di Fabio and Duradoni, 2019). Research also signifies the role of employee proactivity in intrapreneurship (Augusto Felício et al., 2012). In particular, proactivity represents a key employee intrapreneurial competency (Vargas-Halabí et al., 2017). Furthermore, Neessen et al. (2019) aver that employee attitudes such as relation to the organization, satisfaction, motivation, and intention represent key determinants of intrapreneurial behavior.

#### Capabilities

Employees’ educational level relates to their intrapreneurial behaviors (Moriano et al., 2014). Scholars also suggest a number of capability-related characteristics (i.e., skills, abilities, perception of his/her own capability, personal knowledge, and past experience) that determines intrapreneurial behaviors (Stam et al., 2012; Neessen et al., 2019). At an individual level, research finds that employee capabilities such as knowledge (particularly market and technology knowledge) and skills contribute to successful CE initiatives (Bojica et al., 2011). Research considers employee intrapreneurship as a work behavior that is agentic and strategic, aiming to create new ventures or achieve strategic renewal (Gawke et al., 2019). Thus, employees’ innovativeness relates positively to their venture and strategic renewal behaviors. Rigtering and Weitzel (2013) also find that innovativeness can effectively translate employees’ behaviors into intrapreneurial projects. Career adaptability, on the other hand, is the capability to adapt to career challenges smoothly when facing uncertain career environments (Zacher, 2014). Woo (2018) asserts that employee career adaptability affects intrapreneurship; it also mediates the relation between personality traits and intrapreneurship. Based on the Big Five personality dimensions, career adaptability further completely mediates the relation between intrapreneurship and two personality traits: openness and conscientiousness.

#### Judgments

Employee judgment is critical in the process of forming sustainable intrapreneurial initiatives (Melnikas, 2010). Individual level judgment or concern in the present context signifies how an individual forms the intention to join an organization and to play the role of intrapreneur in the organization. Perception of rewards and risks is of particular importance to employee judgments. Gawke et al. (2019) show that reward sensitivity and risk taking relate positively to employee venture behavior and employee strategic renewal behavior. Indeed, employees have several concerns before deciding to participate in intrapreneurial initiatives. These concerns include extrinsic rewards (e.g., financial incentives, opportunities for future growth, etc.) and intrinsic rewards (e.g., feelings of achievement, satisfaction from completing interesting and challenging work, and increased job autonomy, etc.) (Melnikas, 2010; Bojica et al., 2011). Research also suggests that for employees, the most pivotal factors influencing their decision to participate in intrapreneurial activities are the probability of venture success and financial rewards. In contrast, foregone opportunity costs, extra effort requirements, and various risks (job risk, pay risk, and risk of failure) represent major deterrents to employees’ participation (Urban and Nikolov, 2013). Employees take calculated risks in the intrapreneurial process (Sinha and Srivastava, 2013). Hence, employee judgment on risk (both in the face of uncertainty and new challenges) is decisive in the intrapreneurial process (Augusto Felício et al., 2012). Interestingly, Rigtering and Weitzel (2013) indicate that risk taking do not play a role in transforming employees’ behavior into intrapreneurial projects. In addition to the perception of rewards and risks, employees’ organizational identification also leads to their intrapreneurial behaviors (Moriano et al., 2014).

#### Personality Attributes/Traits

Research suggests that employee intrapreneurial competencies comprise several critical personal attributes, such as flexibility and drive (Vargas-Halabí et al., 2017). In fact, personality and dispositional traits represent key factors at the individual level for intrapreneurship, which affect intrapreneurship in various ways (e.g., Stam et al., 2012; Di Fabio et al., 2017; Woo, 2018; Mahmoud et al., 2020). Openness, extroversion, and emotional stability have positive, whereas conscientiousness and agreeableness have negative impact on intrapreneurial behavior (Farrukh et al., 2016). Likewise, de Vries et al. (2016) find that openness, conscientiousness, and extraversion have positive relations with intrapreneurship. Later, Woo (2018) confirms extraversion’s direct effect on intrapreneurship. Nonetheless, the effect of conscientiousness and openness on intrapreneurship is indirect; it is via employee career adaptability. Mahmoud et al. (2020) further show that the following Big Five personality traits, namely, conscientiousness, disagreeableness, and emotional stability relate positively to the intrapreneurial behavior.

As aforementioned, intrapreneurship relates closely to innovation and thus highlights the vital role of engineers within organizations. Based on a longitudinal study of graduate engineers, the results indicate that engineers’ roles and tasks have shifted over time from identifying engineering feasibility to recognizing entrepreneurial opportunities (Solymossy and Gross, 2015), a capability requiring preferable personality traits. Interestingly, these personality traits are more favorable for non-engineers as compared to engineers (Williamson et al., 2013). For example, engineers are generally less open and extravert as compared to non-engineers.

### Enablers-Organizational Level

Organizations, particularly their internal corporate environment such as culture, structure, resources, and communication plays an important role in fostering intrapreneurship. Prior studies identify a list organizational related factors that affecting intrapreneurship. The study summarizes these findings and proposes four major types of enablers at the organizational level: developmental support and work design, resource availability, managerial style, and innovative culture.

#### Developmental Support and Work Design

At the firm level, a supportive structure can promote employees’ intrapreneurial participation (Aramburu and Saenz, 2011; Kuratko, 2017). Research suggests that the organizational structure with management support and work discretion, elicits employees’ intrapreneurial engagement (Neessen et al., 2019). Coaching, as a form of developmental support, represents another promoting factor for employees’ intrapreneurial engagement (Wakkee et al., 2010). Managerial coaching helps intrapreneurs realize why to initiate intrapreneurial activities and what it takes to carry out intrapreneurial projects. Managerial levels provide employees with necessary resources and knowledge through coaching. Acting as knowledge agents who bridge between departments in the organization, managers can also leverage their network and status to stimulate and guide their employees (Wakkee et al., 2010). As such, employees learn to balance their role of intrapreneur with other potentially conflicting roles in existing business operations. Likewise, developmental advice and mentoring help foster intrapreneurial skills and promote sustainable intrapreneurship (Gwynne and Wolff, 2005). On the other hand, Stam et al. (2012) identify important antecedents to intrapreneurship, including job design, work context, and other environmental factors. Among these antecedent factors, job design and work context are essentially important for managers. When firms seek to deploy intrapreneurship within their organizations, organizational policies, and managerial actions exert a direct effect on the above factors.

#### Resource Availability

Resource availability is crucial to employees’ involvement in intrapreneurial activities (Kuratko, 2017; Neessen et al., 2019). Financial resources (e.g., rewards) appear to influence employees’ intrapreneurial attitude (Neessen et al., 2019). Other critical resources range from physical technological systems to invisible intellectual capital of the firm. For example, intrapreneurial workshops represent a useful resource for employees to enhance their skills (Gwynne and Wolff, 2005). Particularly, Rigtering and Weitzel (2013) find that horizontal participation within the firm affect employees’ intrapreneurial behavior. As such, peer-to-peer sharing of knowledge expedites a firm’s venturing process, leading to sustainable intrapreneurial outcomes. Notably, resources such as innovation capability and size effect also enhance a firm’s intrapreneurial engagement (Aramburu and Saenz, 2011).

#### Managerial Style

Rigtering et al. (2019) draw on self-determination theory as well as creativity and framing research to explain how managerial framing affects employees’ engagement in intrapreneurship. Framing pertains to manipulation in the problem formulation or disposition of contextual features in a particular situation. In the stage of intrapreneurial ideation, Rigtering et al. (2019) show that opt-out elicits employee active participation without loss of idea quality. Furthermore, providing hints and offering examples decreases novelty of ideas and the number of idea submissions, but the usefulness of these ideas increases. Plambeck (2012) explores how managerial cognition and organizational factors influence firms’ intrapreneurial actions, and finds that more negative managerial evaluations of the triggering event (e.g., introduction of a new technology) lead to less innovative new products. Furthermore, a firm’s strategy and resources affect the degree of its new product innovativeness and the firm’s sustainability. This effect, however, is partially mediated by executives’ assessment of the triggering event. Organizational research suggests that transformational leadership relates positively to employee intrapreneurial behavior. Transactional leadership, however, relates negatively to employee intrapreneurial behavior (Moriano et al., 2014). As for management support, scholars caution the need to take into consideration the heterogeneity of organizational members (e.g., Hughes et al., 2018). Particularly, Rigtering and Weitzel (2013) find that employee’s trust in the direct manager affect his/her intrapreneurial behavior. Employees’ perception of managerial recognition to their innovative efforts contributes to participation in intrapreneurial activities within organizations (Park et al., 2014).

#### Innovative Culture

A culture of innovation reflects on autonomy and tolerance for failure. Research suggests that autonomy is essential to intrapreneurship (Augusto Felício et al., 2012) or intrapreneurial engagement (Falola et al., 2018). Research also avers that organizational culture that is tolerant of failure are key to intrapreneurial participation (Aramburu and Saenz, 2011). Namely, organizations, if encouraging employees to take risks, can help drive intrapreneurial growth. Organizations also help employees develop intrapreneurial skills and competencies when employees have the opportunity to gather intrapreneurial experience through trial and error. This approach, as a result, leads to an employee perception of his/her enhanced ability and contributes to the intrapreneurial process. Gursoy and Guven (2016) find that firm’s innovative culture affects intrapreneurship positively, on aggregate and by its dimensions (i.e., innovation, risk-taking, proactivity, self-determination, and extension of individual network). Enterprises with organizational culture, which improves market and themselves through innovative and pioneer way to take risk, possess intrapreneurial administration style.

### Facilitating Mechanisms

In addition to the above enablers at individual or organizational level, there exists other facilitators or facilitating mechanisms, which exert effects on the cultivation of intrapreneurial process. As aforementioned, fostering employees intrapreneurial engagement involves several initiatives such as: (1) offering managerial support to initiate innovations, (2) developing, monitoring and implementing novel business ideas, (3) allocating time for strategic brain storming and workshops, (4) designing flexible work system with work discretion liberty, and (5) eliciting employees’ intrapreneurship spirit with appropriate reward systems. Organizations may lose employees with distinctive competencies if they fail to promote and encourage intrapreneurial initiatives within the organization (Armstrong and Taylor, 2014). To support these initiatives, the study summarizes three essential facilitating mechanisms: (1) establishing information and communication technologies (ICT) infrastructures, (2) forming organization–employee relationship (OER), and (3) creating employee–employee relationship (EER).

#### Establishing Information and Communication Technologies Infrastructures

Research finds that implementation of several novel ways of working helps foster intrapreneurial behavior among employees (Gerards et al., 2020). In particular, time- and location-independent work as well as management based on output help promote employees’ intrapreneurial behaviors. Novel ways of working indeed comprises a number of human resource management practices which allow employees to work independent of time and place. Notably, a flexible work environment facilitated by ICT is essential to novel ways of working (de Leede, 2016). As such, rapid advances in ICT (e.g., internet) represent key drivers for the possibility of new ways of working, and serve as a basis for flexible and decentralized working system. Furthermore, ICT-based systems help support the development, monitoring and implementation of new business ideas, which lead to intrapreneurial outcomes.

#### Forming Organization–Employee Relationship

Park et al. (2014) aver that the quality of organization–employee relationship (OER, measured by trust, control mutuality, commitment, and satisfaction) relate positively to employees’ intrapreneurship. Namely, employees are prone to maintain a good relationship with the organization when they feel confident in the organization, and consider their management as competent and reliable, i.e., trust. They perceive their management to be legitimate and to exercise approved power during interactions with them, i.e., control mutuality. They also feel that it is worthwhile to invest in maintaining and promoting the relationship, i.e., commitment. Finally, they feel content with their organization and management as their expectation in the relationship is fulfilled, i.e., satisfaction. Effective OER may also reflect in transformational leadership. Transformational leadership is a leadership approach that leaders inspire their followers, raise their interests, and thus causes change in their followers, with the aim of achieving successful outcomes in the short term or developing followers into leaders in the long term. Research suggests that transformational leadership mediates the relationship between a freely accessible open workplace and intrapreneurial behaviors (Gerards et al., 2020).

#### Creating Employee–Employee Relationship

Forming relationships among employees is critical in the processes for sustainability, innovation, entrepreneurship, as well as intrapreneurship (Chakrabarty, 2020). Research indicates that employees’ involvement and relationships have significant effects on their intrapreneurial engagement (Falola et al., 2018). Specifically, based on self-determination theory (SDT) and relationship-focused theory (RFT), to facilitate the formation of relationships, Chakrabarty (2021) suggests that the interaction between compensation systems (i.e., based on individual or team goal) and the needs for autonomy vs. relatedness among employees corresponds to the type of relationships selected for an entrepreneurial action. Furthermore, Chakrabarty (2021) identify four types of relationships, namely, civil, inspiring, integrating, and synergizing relationships. Accordingly, firms may promote intrapreneurial actions via solitude, solitude with inspiration, integration and with solitude, and synergy approach, respectively. Notably, knowledge sharing also represents a key activity to maintain relationships among employees. Research finds that employee’s knowledge sharing behavior facilitates the intrapreneurial process. Particularly, this type of behavior strengthens the positive relationship between certain types of personality traits (i.e., openness, conscientiousness) and intrapreneurial behaviors (Alam et al., 2020a). Collectively, the study summarizes the above enablers and facilitating mechanisms in Table 2.

**TABLE 2:** Summary of enablers (individual and organizational level) and facilitating mechanisms for cultivation of intrapreneurship.

**Enablers-individual level**	**Enablers-organizational level**	**Facilitating mechanisms**
*Self-attitudes*	*Developmental support and work design*	*Establishing information and communication technologies (ICT) infrastructures*
• Personal initiative	• Management support	ICT-based systems help support:
• Entrepreneurial self-efficacy	• Work discretion	• Novel ways of working (time- and location-independent work)
• Proactivity	• Managerial coaching	• Flexible and decentralized working system
*Capabilities*	• Developmental advice and mentoring	• Ideas development
• Market knowledge	• Job design and work context	• Monitoring intrapreneurial process
• Technology knowledge	*Resource availability*	• Implementing new business ideas
• Innovativeness	• Financial resources (e.g., rewards)	*Forming organization–employee relationship (OER)*
• Career adaptability	• Technological systems	OER reflects on:
*Judgments*	• Intrapreneurial workshops	• Trust
• Rewards (finance, achievement, and satisfaction)	• Related knowledge	• Control mutuality
• Risks (job, pay, and reputation)	• Innovation capability	• Commitment
• Probability of venture success	*Managerial style*	• Satisfaction
• Organizational identification	• Managerial framing	*Creating employee–employee relationship (EER)*
*Personality attributes/traits*	• Transformational leadership	• Civil relationship
• Flexibility and drive	• Managerial receptiveness	• Inspiring relationship
• Openness	*Innovative culture*	• Integrating relationship
• Conscientiousness,	• Autonomy	• Synergizing relationship
• Extroversion	• Tolerance for failure	• Knowledge sharing among employees
• Emotional stability	• Encouraging risk-taking	
	• Extension of individual network	

## Challenges

Intrapreneurship undoubtedly helps firms attain financial performance, productivity, as well as sustainable growth and improvement. Exploring the important factors that drive successful intrapreneurship, as a result, has drawn abundant research attention. The pursuit of those antecedent factors and conditions to foster intrapreneurship, however, brings forth several challenges for firms. The study highlights these challenges arising from both managerial and technological aspects. They are delineated as follows.

### Managerial Challenges

The study identifies three main managerial challenges, which arise from a firm’s capability in (1) fostering an intrapreneurial culture, (2) designing suitable work relationship, and (3) offering appropriate incentives. First, managerial support is essential for building an intrapreneurial culture within an organization. Intrapreneurial culture sustains the organization’s development through exploring talented employees and promoting intrapreneurial activities that foster innovation within the organization (Lukes and Stephan, 2017). Early intrapreneurial research posits that businesses may not have the organizational climate and culture to nurture intrapreneurs even if they have abundant resources (Duncan et al., 1988). This phenomenon has been improved as a growing number of firms start to realize the importance of intrapreneurship on innovation and sustainability. Still, several challenges for organizations to stimulate intrapreneurial activities remain largely unchanged over the past decades. For example, large organizations usually are not appropriate environments to nurture intrapreneurship (Sharma and Chrisman, 1999). Likewise, contention between employee and the managerial level signifies a primary reason why many intrapreneurs leave the organizations and start their own businesses (Klepper, 2001). Above all, employees with intrapreneurial spirits may have started their own businesses (i.e., became entrepreneurs) before being hired by others.

Second, as noted above, drawing on SDT and RFT, firms may facilitate the intrapreneurial process with appropriate choices of working relationships. Nonetheless, there exists apparent concerns and issues in the implementation of compensation systems, namely, fairness of an allocation under individual based compensation as well as free-riding problems under team-based compensation. The implementation of compensation systems and the type of relationships chosen for intrapreneurial action evidently influence a team’s intrapreneurial performance.

Finally, being paid employees rather than real entrepreneurs, employees are unlikely to take the risk and responsibility associated with intrapreneurship. Intrapreneurial initiatives often encounter barriers to motivating employee participation (Gibbs et al., 2017). Indeed, firms already strived to offer employees motives to involve in conventional suggestion systems that solely solicit idea submission (Gibbs et al., 2017). Furthermore, the output quality of intrapreneurship (e.g., the novelty and usefulness of generated ideas) represents another concern of managers (Perry-Smith and Coff, 2011). Based on SDT, merely resorting to intrinsic task motivation is inadequate to involve paid employees in intrapreneurship. Adopting social norms as an external stimulus, Rigtering et al. (2019) aver that automatic registration (i.e., participating in a CE initiative out of concern for the implied social norm, but opt-out is allowed) encourages employee participation without quality loss in ideas. Interestingly, providing examples decreases the novelty and number of idea submissions but the proposed ideas seems to be more useful. Still, research largely agrees that when stimulating creative performance such as novelty and usefulness, intrinsic stimuli are more appropriate than extrinsic ones. Hence, designing an intrapreneurship initiative to effectively motivate employees remains a challenge for firms.

### Technological Challenges

To foster intrapreneurship, the study points out various organizational-level factors and concerns which lead to managerial challenges. In addition to the managerial challenges, several technological challenges exist. Academics and practitioners commonly agree that, technological, particularly digital transformation offers organizations opportunities to involve intrapreneurs. Ironically, while firms constantly urge the need to generate and collect employees’ ideas, in reality firms often lack tangible and IT-based tools and processes to efficiently leverage these ideas into intrapreneurial projects and innovative products. These technological challenges range from building an isolated IT system to facilitate the internal intrapreneurial process, to constructing a sophisticated digital intrapreneurship platform that connects external resources.

For example, merely building an entrepreneurially alert information system may impart a positive effect on firm’s intrapreneurial performance (Simsek et al., 2009). Nonetheless, it becomes much more challenging when developing a digital intrapreneurship platform as the platform inherently represents a “socio-technical system”, which recognizes and underlines the interaction between people and technology in the workplace (Reibenspiess et al., 2020). Entrepreneurial research avers that entrepreneurs can employ the platform-based innovation ecosystem to facilitate the entrepreneurial process (Hsieh and Wu, 2019). Furthermore, Hsieh and Wu (2019) suggest a specific type of open intrapreneurial platform for firms particularly with high commercialization and new product/service development (NPD/NSD) capabilities. “New economy” conglomerates such as Alphabet and Proctor and Gamble nurture their internal ventures via this type of platforms. Even though firms commit to offering needed resources for their intrapreneurs, quite a few large enterprises (e.g., Siemens and GE, etc.) also engage with external partners (i.e., other organizations or institutes) via the open intrapreneurial platforms. Notably, these digital platforms are often found within large enterprises. Developing such an IT infrastructure apparently remains a challenge for firms with less commercialization and NPD/NSD capabilities.

## Future Research Agenda

The study provides insights into various facets through which organizations can better design and cultivate intrapreneurship. These discussions also raise several concerns and challenges, which point to certain research topics worth further exploration. The study proposes several future research opportunities as follows.

### Interlocking Between Individual and Organizational Factors

Within organizations, intrapreneurship is multifaceted encompassing the behaviors and interactions of the individual, organizational, and other environmental elements. Various levels (i.e., top-, middle-, and operational-level) of managers and employees play specific roles in the intrapreneurial process. Top and middle level managers generally focus on opportunity recognition. They are also expected to develop a strategic vision on intrapreneurship and manage the intrapreneurial process (Ireland et al., 2009). Operational-level managers and employees, as a major source of new ideas, however, are often responsible for implementation (Anderson et al., 2014). Hence, they usually play a dual role both in recognizing and exploiting intrapreneurial opportunities, and instigate renewal and innovation within the firm. Employees’ intrapreneurial creativity and domain knowledge serve as a basis for ideation (Liu et al., 2016; Rigtering et al., 2019), whereas opportunity recognition relates to specific dynamics at the dyad, group, and organizational level (Grégoire et al., 2010). Namely, the extent to which employees may unleash their creativity for opportunity recognition depends on several factors such as organizational design, how firms organize their intrapreneurial processes, and interactions among individuals in the organization (Corbett et al., 2013). To date, the above research areas still receive insufficient attention and thus merit further examination.

### Roles of Demographical and National Factors in Intrapreneurship

Extant research reports mixed results of the impact of demographical factors on intrapreneurship. For example, Moriano et al. (2014) suggest that higher educational level achieved by employees leads to more intrapreneurial behaviors. As such, well-educated employees are often competent and innovative, allowing them to initiate more intrapreneurial projects. Nonetheless, Douglas and Fitzsimmons (2013) find that employees with doctoral degrees are less likely to form intrapreneurial intentions, whereas bachelor and master’s degrees do not relate to intrapreneurial intentions. Regarding personality traits, de Vries et al. (2016) and Mahmoud et al. (2020) find a positive, while Farrukh et al. (2016) observe a negative impact for conscientiousness on intrapreneurial behavior. Across countries, Thai citizens are less inclined to engage in intrapreneurship compared to the Australians. Further studies exploring the above contradictory or interesting phenomena shall be worthwhile.

### Types of Interactions Among Employees at Various Intrapreneurial Stages

Social interaction allow intrapreneurs to collect valuable information, identify innovative opportunities, and persuade colleagues to support their initiative in the organization (Gerards et al., 2020). Research suggests that social interaction among employees supports the creation of a knowledge network for developing intrapreneurial ideas (e.g., Castrogiovanni et al., 2011). Particularly, employee’s knowledge sharing behavior facilitates the intrapreneurial process (Alam et al., 2020a). Nonetheless, concerns about the appropriate types of interactions occurred at the corresponding stages of the intrapreneurial process still remain underexplored.

### Intrapreneurship for Small and Medium-Sized Enterprises in the Digital Era

As noted above, large firms may utilize the innovation ecosystem, which is based on platforms, in the intrapreneurial process according to their commercialization and NPD/NSD capabilities (Hsieh and Wu, 2019). In contrast, SMEs typically lack necessary operational and technical resources to support their intrapreneurial process. Despite organizations’ efforts to exploit the innovation potential of their employees, for SMEs, infrastructures such as digital intrapreneurship systems or platforms facilitating this innovation process are deficient. As such, employees may demonstrate the zeal and behaviors of being intrapreneurial, but innovating within organizational boundaries with the help of digital support is unlikely. Thus, future research may probe into the design of viable platforms facilitating intrapreneurial behavior particularly for SMEs.

### Assessment and Integration of Focused and Dispersed Approaches

Focused and dispersed approaches to foster intrapreneurship within firms apparently require different design of managerial mechanisms, such as work, rewards system, and performance review, etc. While a stream of research emphasizes the advantage of adopting the dispersed approach (e.g., Belousova and Gailly, 2013), these studies do not take into consideration several crucial factors (e.g., the size of the firm, nature of the business, and type of the industry). Future research may evaluate the differences between these two approaches in detail. Furthermore, inspired by the matrix organizational structure, researchers may even consider a combined approach which claims benefits from both the focused and dispersed approaches.

### Contextual Examination of Risk Propensity

As aforementioned, employee judgment on risk is crucial in the intrapreneurial process. Nonetheless, prior research reports inconsistent findings. For example, Boon et al. (2013) note that both entrepreneurs and intrapreneurs are proactive, innovative, and willing to take risks, as expected. Specifically, intrapreneurs are generally reluctant to take financial risks such as the risk of losing their jobs, or the risk of being responsible for the financial losses of their firms. Instead, intrapreneurs are more willing take reputational risks such as voicing their opinions and ambitions, or initiating a complex project. Interestingly, Rigtering and Weitzel (2013) indicate that innovativeness and personal initiative, rather than risk taking, convert employees’ behavior into intrapreneurial projects. The role of risk seems to be conditional on contextual factors such as intrapreneurial stage, leadership style, etc. (e.g., Bagheri and Pihie, 2011), which warrants future research.

### Intrapreneurial Roles Across Organizational Levels

Research emphasizes the need to examine how intrapreneurship contributes to a firm’s development and why some organizations experience successful deployment in intrapreneurship at every hierarchical level (Wales et al., 2011). Belousova and Gailly (2013) show that various levels (i.e., top-, middle-, and operational-level) of managers play particular roles in the corporate entrepreneurial process at different stages (i.e., discovery, evaluation, legitimation, and exploitation stage). Specifically, middle level managers are critical as they stimulate operational-level employees to generate innovative ideas; meanwhile, they also need to champion ideas as part of their managerial duties. Some studies argue that under certain circumstances, senior managers in the organization possess more power to leverage the resources and thus initiate more intrapreneurial activities than first-level managers do (Urban and Nikolov, 2013). Intrapreneurial roles at different organizational levels appear to associate with some organizational factors which deserve further studies.

### Exploration Versus Exploitation in the Pursuit of Intrapreneurship

Evidently, nurturing intrapreneurship helps revive organizations, allowing them to adapt to external changes and achieve innovations and sustainability. From the resource-based view, intrapreneurship serves as a valuable strategic resource that enable firms to fit to their external environment. Research identifies exploitation and exploration as two pivotal approaches for firms to adapt to the environment (e.g., Skarmeas et al., 2016). Selecting between the approaches, however, needs to take into consideration the types of intrapreneurial opportunity based on strategic importance to the firm and the firm’s existing resources (Corbett et al., 2013). Even if firms may adopt the ambidextrous approach (i.e., coexisting of exploration and exploitation) when the intrapreneurial opportunity demonstrates strategic importance and capitalizes on the firm’s existing assets (O’Reilly and Tushman, 2008), it is usually difficult for firms to master exploration and exploitation simultaneously. Hence, future research may investigate the circumstances under which firms’ intrapreneurial initiatives are better suited for exploration or exploitation.

## Conclusion

Over the years, intrapreneurship has drawn increasing attention from both academics and practitioners. The growing interests attribute to two important values intrapreneurship may bring about: (1) intrapreneurship as an approach to achieve innovations and business sustainability, and (2) intrapreneurship as a principle guiding businesses to adapt to environmental changes and enhance their performance. Intrapreneurship transforms the way business pursue innovations and sustainability, affecting both businesses and their employees. The prevalence of intrapreneurship creates opportunities for businesses. On the other hand, it also poses challenges for them. The study reveals how intrapreneurship relates to other prominent theoretical concepts: entrepreneurship, innovation, and sustainability in the context of business management. The study also proposes a conceptual framework that encompasses both the enablers (at individual and organizational levels) and facilitating mechanisms, based on which, firms may cultivate intrapreneurship. Thereby, the study discusses the challenges and raises several research opportunities.

Theoretically, this study contributes to the existing literature in that it examines intrapreneurship through various theoretical lens of innovation, entrepreneurship, and sustainability. As such, the discussion paves the way for developing a theoretically parsimonious intrapreneurial framework in relation to these emerging business initiatives. The study also represents an early effort to provide a holistic view incorporating key enablers both at the individual and organizational levels and facilitating mechanisms to nurture intrapreneurship. Moreover, this study proposes a number of research topics. Pursuit of these opportunities shall provide a valuable addition to the sparse intrapreneurial literature. Managerially, organizations can better design their work systems and comprehend the needs from employees in the intrapreneurial process based on the present study. Furthermore, the described concerns and issues help managers at various levels ameliorate the intrapreneurial climate within organizations. Employees also learn from this study by understanding pivotal factors shaping their behaviors in the intrapreneurial process.

## Author Contributions

L-YH, S-MY, and Y-JH contributed to conception and design of the study and provided suggestions to all sections of the manuscript. L-YH organized the research framework. S-MY performed the literature analysis and comparison. Y-JH wrote the first draft of the manuscript. All authors contributed to manuscript revision, read, and approved the submitted version.

## Conflict of Interest

The authors declare that the research was conducted in the absence of any commercial or financial relationships that could be construed as a potential conflict of interest.

## Publisher’s Note

All claims expressed in this article are solely those of the authors and do not necessarily represent those of their affiliated organizations, or those of the publisher, the editors and the reviewers. Any product that may be evaluated in this article, or claim that may be made by its manufacturer, is not guaranteed or endorsed by the publisher.
